# Letter to the Editor: Is paliperidone palmitate more effective than other long-acting injectable antipsychotics?

**DOI:** 10.1017/S0033291718001319

**Published:** 2018-06-04

**Authors:** Mitesh Desai, Annette Wooller, Arunas Lignugaris, Srihari Gopal

**Affiliations:** 1 Janssen-Cilag Limited, High Wycombe, UK; 2 Janssen EMEA, High Wycombe, UK; 3 Janssen Research & Development, LLC, Titusville, NJ, USA

We read with interest the recent paper by Patel *et al.* (2017) exploring the effectiveness of long-acting injectable antipsychotics (LAI) and the authors’ conclusion in the main body of the paper that: ‘this study suggests that paliperidone palmitate was at least as effective as other LAI antipsychotics. A key issue to address in future studies is whether paliperidone is more effective than other LAIs when given to patients who are matched for illness severity and prognosis.’

Mirror-image studies are frequently used to evaluate the comparative effectiveness of antipsychotic medicines as they reflect real-world utilisation and outcomes that cannot be elucidated from classical randomised control trials; furthermore, they offer the advantage of each participant serving as his/her control (Kane *et al.*, 2013). In their analysis of a large population (*N* = 1281), Patel *et al.* (2017) found that in the 3 years prior to initiating paliperidone palmitate (1-monthly maintenance, PP1M), individuals had a statistically significant, greater number of hospitalisations and bed days compared with those initiating other LAIs. Since the number and length of prior hospitalisations is believed to be a valid surrogate for the number and severity of prior relapses, based on Lieberman *et al.* (2001), individuals in the PP1M group probably had more severe disease and as such, their potential for recovery would be impaired compared with those on other LAIs. However, over the subsequent 3 years of follow-up, hospitalisations were similar between the PP1M and the other LAI groups. Thus, achieving comparable outcomes despite a reduced potential for recovery could more likely suggest that PP1M has greater efficacy, rather than ‘not being more effective’ than other LAIs, including those that may be cheaper.

In their critique of comparative research in psychiatry, Kane *et al.* (2013) identify expectancy bias and changes in service provision and utilisation as some of the potential confounding factors in naturalistic, mirror-image studies. Based on the audit of prescribers, Patel *et al.* (2017) report PP1M to be the most frequently prescribed LAI with respondent comments suggesting benefits of PP1M above other LAIs including efficacy and side effect profile.

In relation to disease severity, could prescribers be preferentially using PP1M when patients are acutely psychotic and/or have had multiple prior relapses, as suggested by 60% commencing PP1M as an in-patient at the same healthcare institute (Taylor *et al.*, 2016)? Additionally, as PP1M can be initiated without prior oral stabilisation in individuals with mild/moderate schizophrenia who have confirmed responsiveness and tolerance to risperidone/paliperidone (Janssen-Cilag International N.V., 2017), and without the need for additional oral antipsychotic supplementation, is PP1M being used to to attain rapid therapeutic levels to permit earlier discharge? Such considerations are important because, with the mirror-image point (index date) defined as the date of the first LAI prescription plus 1 month, Patel *et al.* (2017) may have potentially underestimated the impact of PP1M given that the bed days avoided in the first 30 days would not have been counted. Without further sensitivity analyses to overcome inter-antipsychotic variability based on dose, dose frequency and pharmacokinetic profile of individual agents, it would be difficult to understand this fully.

Patel *et al.* (2017) did acknowledge the importance of treatment continuation in their reference to a real-world study that found greater treatment continuity with PP1M (Decuypere *et al.*, 2017); and an open-label clinical trial which failed to demonstrate a clinically meaningful difference in its primary endpoint or to show a statistical difference in discontinuation rates between PP1M and the comparator (Naber *et al.*, 2015). However, despite the significant importance of treatment discontinuation as a risk for hospitalisations (Weiden *et al.*, 2004), Patel *et al.* (2017) did not assess treatment continuation as group allocation was time censored based on the first injection as recorded in the electronic health records. Therefore, this represents incidence use of LAIs and not ‘prevalence’ as indicated in the paper. This significant limitation could be overcome by assessing outcomes such as hospitalisation and or treatment failure in relation to total drug exposure for different drugs and/or preparations (oral *v.* LAI) (Tiihonen *et al.*, 2017).

In summary, we are in agreement with the conclusion in the main body of the paper, in contrast to the abstract, that PP1M was at least as effective as other antipsychotics and a key issue to address in future studies is whether paliperidone palmitate is more effective than other LAIs when given to individuals who are matched for illness severity and prognosis. Importantly, we believe this conclusion accurately reflects findings of the study as: (1) it appropriately considers an important clinical and statistical difference at baseline between the PP1M and other LAI groups, i.e. difference in prior hospitalisations; (2) highlights the need to control for the important clinical difference in future comparative research; and (3) would still take into account the limitations of the study design for coming to robust conclusions.
